# Classifying Changes in Amputee Gait following Physiotherapy Using Machine Learning and Continuous Inertial Sensor Signals

**DOI:** 10.3390/s23031412

**Published:** 2023-01-27

**Authors:** Gabriel Ng, Jan Andrysek

**Affiliations:** 1 Institute of Biomedical Engineering, University of Toronto, Toronto, ON M5S 1A1, Canada; 2 Bloorview Research Institute (BRI), Holland Bloorview Kids Rehabilitation Hospital, Toronto, ON M4G 1R8, Canada

**Keywords:** gait classification, inertial sensors, machine learning, rehabilitation, lower limb amputees, time-series analysis

## Abstract

Wearable sensors allow for the objective analysis of gait and motion both in and outside the clinical setting. However, it remains a challenge to apply such systems to highly diverse patient populations, including individuals with lower-limb amputations (LLA) that present with unique gait deviations and rehabilitation goals. This paper presents the development of a novel method using continuous gyroscope data from a single inertial sensor for person-specific classification of gait changes from a physiotherapist-led gait training session. Gyroscope data at the thigh were collected using a wearable gait analysis system for five LLA before, during, and after completing a gait training session. Data from able-bodied participants receiving no intervention were also collected. Models using dynamic time warping (DTW) and Euclidean distance in combination with the nearest neighbor classifier were applied to the gyroscope data to classify the pre- and post-training gait. The model achieved an accuracy of 98.65% ± 0.69 (Euclidean) and 98.98% ± 0.83 (DTW) on pre-training and 95.45% ± 6.20 (Euclidean) and 94.18% ± 5.77 (DTW) on post-training data across the participants whose gait changed significantly during their session. This study provides preliminary evidence that continuous angular velocity data from a single gyroscope could be used to assess changes in amputee gait. This supports future research and the development of wearable gait analysis and feedback systems that are adaptable to a broad range of mobility impairments.

## 1. Introduction

For individuals with lower-limb amputations (LLA), efficient and purposeful rehabilitation is crucial for developing healthy movement strategies, reducing the risk of long-term musculoskeletal issues, and improving quality of life [1,2,3]. Current rehabilitation practice is largely subjective, which significantly impacts our ability to reliably monitor and improve gait over time [4,5]. Various methods and technologies have been developed for quantitative gait and movement analysis [6]. Wearable technology, and in particular inertial sensors, offers the possibility of cost-effective, portable, and clinically viable tracking of human movement and gait, with application to such areas as gait event detection [7,8,9], postural balance and control [10,11] calculation of spatiotemporal and kinematic gait parameters [12,13,14], and machine-learning-based gait classification [15,16,17]. Research for LLA focuses primarily on gait parameter assessment and gait event/gait cycle detection [7,8,18,19], but wearable systems for gait classification could play an important role in rehabilitation and research realms [12,20,21] for LLA and general gait disabilities. For example, they could inform clinicians and patients about changing gait patterns over time and whether gait patterns are improving towards some desired rehabilitation targets or are worsening and are thus in need of further intervention (e.g., physiotherapy).

Several research groups have successfully applied ML to gait classification, specifically focusing on continuous signals rather than using discrete gait parameters [15,16,22,23]. Continuous signals, in this case, refer to time-series data directly from a sensor or of a kinematic curve as opposed to discrete, summarized parameters, which typically refer to a single value. While many studies have investigated classification based on gait parameters [20], using time-series for ML classification offers certain advantages compared to discrete parameters. It eliminates the need to predefine an extensive set of parameters and feature extraction techniques [24]. This can be a challenge for designing compact wearable systems such as inertial sensors, which may require multiple sensors at various locations to measure different spatiotemporal and kinematic parameters [14,25,26]. Time-series can also capture time dependence in human movement and gait that discrete parameterization cannot [27]. Work on time-series classification has largely employed person-specific models [15,22,23]. Person-specific classification differs from population-based models in that models are trained and tested on single individuals rather than grouped populations. While population-based techniques can simplify model training requirements and offer generalizable diagnostics (e.g., healthy vs. Parkinson’s, youth vs. geriatric population, etc.) [20], person-specific models may be necessitated when dealing with highly heterogeneous populations (such as in LLA rehabilitation) and in accurately classifying more subtle gait changes over time. One common application of person-specific models has been for the classification of an individual’s fatigued vs. their non-fatigued gait [15,22]. Baghdadi et al. used Euclidean distance metrics combined with a nearest-neighbor classifier to classify pre- and post-fatigued gait based on time-series data from a foot-mounted inertial sensor. Janssen et al. used the continuous kinetic signals of ground reaction forces and a self-organizing map model to classify between pre- and post-fatigued gait [22]. Additionally, Wang et al. used dynamic time warping (DTW) to compare the kinematic curves of individuals with Parkinson’s before and after medication [23]. Continuous signals have been demonstrated to be well-suited to capture inter- and intra-individual gait characteristics and changes [22,28] and could capture changes in gait independent of specific gait parameters.

To the best of our knowledge, no study has investigated the use of these techniques in LLA or in the context of providing training to alter an individual’s gait. In this exploratory feasibility study, we aimed to use ML techniques to accurately classify an individual’s gait before and after a session of physiotherapy. Additionally, unlike previous literature which uses time-series for gait classification, we proposed a method that uses only the angular velocity data from a single inertial sensor for classifying between the two gait states. The use of a single sensor (or minimal set of sensors) is motivated by an end goal, to ultimately develop a simple-to-use, cost-effective wearable gait assessment system. Further, the testing paradigm (i.e., classifying gait changes related to physiotherapy) aligns well with the clinical practice since LLA and lower-limb impairment typically involve gait training with a physiotherapist (PT) [29] to improve gait dynamics. Finally, to better understand the performance of our model, we compared results with gait changes which were assessed using conventional gait parameters and measured with a commercial gait analysis system.

## 2. Materials and Methods

### 2.1. Participants

Five LLA were recruited (three males and two females). The median age was 13, with participants ranging from 9 to 19 years old. Table 1 details individual participant demographics. There was one bilateral transtibial amputee and four unilateral amputees: one transtibial, two Van Nes rotationplasty cases, and a user with a locked orthotic brace affixed at the hip to address limb shortening. Participants were independent ambulators, and only P1 used an assistive device (cane) during the experimental session. The recruitment and experimental procedure were approved by the Research Ethics Board at Holland Bloorview Kids Rehabilitation Hospital (REB-0176). All participants were assessed as having the capacity to consent and provide written consent prior to their participation. These participants were recruited by the prosthetist or physiotherapist in their circle of care and were either receiving physiotherapy sessions already at the hospital or were identified as having the potential to benefit from physiotherapy targeting gait training.

Five able-bodied participants were also recruited (two female, three male; median age 25, ranging from ages 23 to 27 years). Able-bodied participants were included if not exhibiting any obvious gait abnormalities (self-assessed) and did not have any prior history of cardiovascular, neurological, or musculoskeletal disorders.

### 2.2. Instrumentation, Protocol and Data Acquisition

#### 2.2.1. Instrumentation

**Figure 1:**
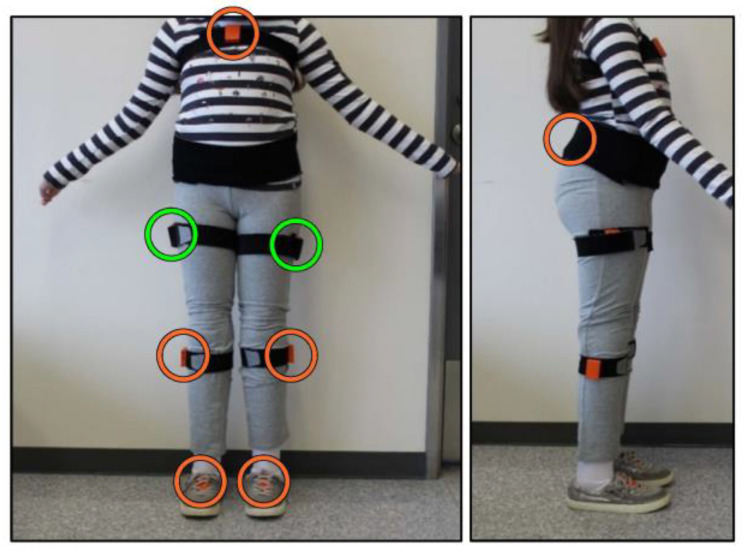
Example participant setup for the Xsens Awinda. Sensors on bridge of both feet, lower legs below the knee (shank), outside of upper legs parallel with sagittal plane (thigh), center of pelvis on the sacrum, and center of sternum. The upper leg sensors are highlighted in green. Data from only one of the upper-leg sensors used for ML model (prosthetic side for unilateral amputee, left side for bilateral amputee).

All participants were instrumented using the Xsens Awinda system (Xsens Technologies BV, Enschede, The Netherlands) with eight inertial sensors located on the lower body and sternum (Figure 1). Sensors were placed on firmly secured Velcro straps. The placement of the sensors followed the anatomical markers suggested in the Xsens user manual [30], and the sensors were worn throughout the whole protocol, including during gait training. Xsens Awinda is well-validated against gold-standard optical motion capture systems and is regarded as a gold standard for inertial measurement systems [31,32,33]. It collects accelerometer, gyroscope, and magnetometer data (all at 100 Hz) to measure the linear and rotational movements at the sensor locations. It also reports foot-contact information and joint angles, which were used to calculate spatiotemporal and kinematic gait parameters. However, only the gyroscope data from a single sensor on the upper leg of the prosthetic side was used for the development of machine-learning models. This location was chosen based on previous work [12,34].

#### 2.2.2. Data Collection Protocol

**Figure 2:**
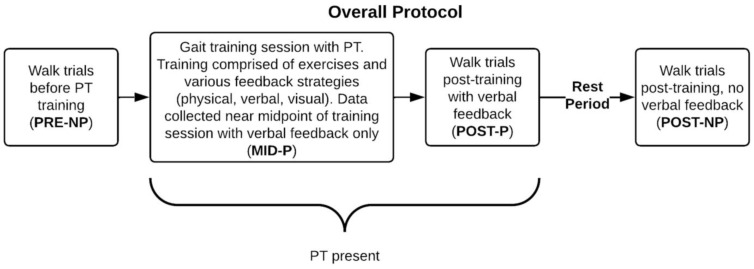
Overall data collection protocol.

The protocol was centered around a session of gait training led by a PT (two PTs were involved in the study), and LLA participants completed four sets of walking trials: before the PT gait training session (PRE-NP), near the midpoint of the training (MID-P), immediately following the training (POST-P), and after a brief 10–15 min rest period (POST-NP). Trials with the -NP abbreviation indicate no verbal feedback from the PT, while -P means that the PT provided verbal feedback (and no other type of feedback, e.g., physical or visual) during the walking trial. Verbal feedback involved reminders of what was worked on during the session, such as muscle activation, mental visualization cues for the participant, gait timing, etc. For each set of walking trials, participants completed at least 10 passes on a 20 m straight path to collect 100–125 strides with the wearable gait analysis (Xsens) system. The number of strides was based on papers that also used time-series for gait classification [15,22]. Figure 2 shows the overall protocol.

Following PRE-NP, participants received physiotherapy from a certified PT lasting 45–60 min. The physiotherapy involved the participants working with the PT through various training exercises/drills to address core functional or mobility goals (such as increasing weight-bearing on the prosthetic leg, improving the smoothness of the gait cycle, etc.) as determined by the PT. The PT provided physical, verbal, and visual feedback to achieve the desired goals and targeted gait improvements. Typically, gait training sessions began with a mix of feedback strategies (physical, verbal, and visual) and progressed to mostly verbal feedback before continuing to reinforce the gait patterns that the participant had been working on. PRE-NP and POST-P aimed to capture baseline gait and post-physiotherapy gait, respectively. MID-P and POST-NP were collected to assess changes during the session (once the session had progressed to predominantly verbal feedback from the PT) and short-term retention or regression following the session, respectively.

We also collected data from able-bodied participants to obtain datasets from participants whose gait did not change during the experimental protocol. Able-bodied participants wore an ankle weight (5 lbs.) throughout the session on their non-dominant ankles to simulate an atypical/asymmetric gait pattern [35]. They completed the same protocol as the LLA participants, except instead of receiving a session of gait training (Box 2, Figure 2), participants completed 30 min of walking (i.e., no intervention was given to change their gait during the session).

### 2.3. Model Development and Evaluation

**Figure 3:**
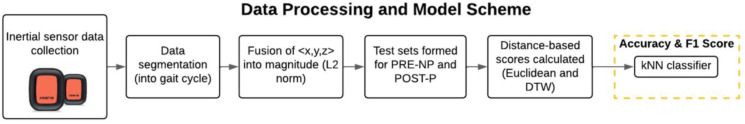
Flowchart of model for classifying pre- and post-training gait using continuous signals from inertial sensors.

The methodology was implemented with code developed in Python 3.8.1. This included model implementation and evaluation, as well as calculating gait parameters from Xsens xml data and performing statistical tests on gait parameters. The overall data processing and classification scheme is shown in Figure 3.

#### 2.3.1. Data Preprocessing

Prior to inputting the data from the gyroscope into the ML models, the raw signals were partitioned into time-series representing individual gait cycles (foot-strike to foot-strike) using foot contact data from the Xsens Awinda system. The strides were resampled to normalize them with respect to time (i.e., all strides are the same vector length). This is both for simplicity feeding into the machine learning models as well as to reduce the effects of cadence/speed and focus the models on the overall signal shape/profile. Finally, we computed the magnitude of the angular velocity using Equation (1). This combines the three axes (*x*, *y*, *z*) into a single signal for input into our models.

(1) $$\left|v_{angular}\right|=\sqrt{v_{xi}^{2}+v_{yi}^{2}+v_{zi}^{2}}\;\;\;\;\;\;for\;each\;i\;in\;v_{angular}$$

#### 2.3.2. Model Development

Our model aimed to classify the PRE-NP and POST-P gait strides using the partitioned time-series data from the inertial sensor gyroscope. Different methods exist for time-series analysis and pattern distinction. These have been most commonly applied to gesture recognition and, more recently, to gait pattern recognition [15,22]. We utilized dynamic time warping (DTW) for its ability to achieve high accuracy with smaller datasets and less training to learn model parameters. We tested both Euclidean distance and DTW metrics. These are two of the most common metrics for time-series comparisons, which, even given their relative simplicity, are still exceedingly powerful metrics [36]. For Euclidean distance, we used a normed version which is shown in Equation (2). This calculates the root mean squared error between the two signals.

(2) $${\left||x-y|\right|}_{2}=\sqrt{\frac{\sum {\left(x_{i}-y_{i\;}\right)}^{2}}{n}},\;n=length\;of\;vectors$$

**Figure 4:**
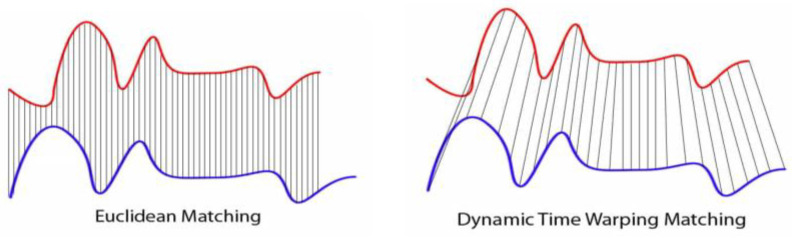
Graphical representation of Euclidean distance matching vs. dynamic time warping (DTW). For Euclidean matching, distances are calculated between corresponding indices only. For DTW, points are matched with their closest analog to minimize distance between the two time-series.

DTW differs from Euclidean distance in that for a given point in a signal, it finds the closest matching point, allowing for some warping of the signal. Every index from the first sequence must be matched with a point from the second, and the mapping must be monotonically increasing, i.e., if *j* > *i* are indices from the first sequence, then there must not be two indices *l* > *k* in the other sequence, such that index *i* is matched with index l and index *j* is matched with index *k*, and vice versa). This is demonstrated in Figure 4.

Given two matrices X = [*x*_1_, *x*_2_, …, *x_n_*] and Y = [*y*_1_, *y*_2_, …, *y_m_*], DTW builds a distance matrix *D* such that *D*(*i,j*) is the distance between points X(*i*) and Y(*j*). A matrix Θ is then constructed where:

(3) Θ(i, j)=D(i,j)+min[Θ(i−1, j−1), Θ(i−1, j), Θ(i, j−1)]

Each element of Θ thereby contains the optimal distance between the vectors [*x*_1_, …, *x_j_*] and [*y*_1_, …, *y_j_*], and the final element of Θ is the minimum global error between vectors X and Y which are reached by minimizing the local errors between the two sequences. Various metrics can be used to compute the distance between the points, *D*(*i,j*); in this work, squared distance was used for computing local errors for DTW. We also imposed a fixed window size which limits the amount of warping by the algorithm (i.e., how far apart in the signal points can be matched). This was chosen empirically based on preliminary accuracy testing.

#### 2.3.3. Model Evaluation

An equal number of strides were used from the PRE-NP and POST-P trials. A subset of 20% for strides from both PRE-NP and POST-P were used as test data, with five-fold cross-validation performed. This was conducted on a participant-specific basis (i.e., participant strides were not grouped together). Each of the strides in the test sets was compared to the training data (PRE-NP and POST-P train sets) to produce distance scores using both the Euclidean and DTW metrics. These were used as the lone feature for a k-nearest-neighbor (kNN) classifier to classify test strides as PRE-NP or POST-P. This follows the approach of similar papers employing the nearest-neighbor and/or distance-based classification strategy [15,16,37]. The performance of the classifiers was evaluated based on prediction accuracy, with the reported accuracy being the average of the cross-validation. The MID-P and the POST-NP strides were also used as model input. These were classified as either PRE-NP or POST-P to assess whether classification correlated with the changes in gait parameters (see Section 2.4). Specifically, to determine the model’s clinical relevance, we wanted to assess whether these strides would be classified as the trial that more closely matched its target gait parameters. Model testing and accuracy results, as well as gait parameter analysis, were performed on a person-specific basis as well.

Performance was assessed for both the LLA and able-bodied participants. This was to compare the model’s accuracy by classifying between PRE-NP and POST-P trials for LLA participants receiving gait training with participants whose gait did not significantly change during the data collection protocol (i.e., able-bodied).

### 2.4. Gait Parameter Extraction

#### 2.4.1. Common Gait Parameters (Identifying Changes between PRE-NP and POST-P)

To determine whether a participant’s gait changed significantly between PRE-NP and POST-P, a selection of common gait parameters was calculated for each of the participants. Foot contact (i.e., heel-strike and toe-off events) and position data from the Xsens were used to calculate spatiotemporal parameters (definitions of spatiotemporal parameters in relation to gait cycle contact events [38,39]). Joint angle minima/maxima were obtained from the kinematic data to calculate kinematic parameters. These parameters were: stance-time symmetry ratio, double-support stance percentage, stance time (prosthetic/non-prosthetic side), step length (prosthetic/non-prosthetic), knee flexion/extension range of motion (prosthetic/non-prosthetic), and hip flexion/extension range of motion (prosthetic/non-prosthetic). Stance-time symmetry was chosen because it is a common gait parameter for LLA [40,41]. Knee and hip flexion/extension were measured since we anticipated that upper thigh gyroscope data (and therefore classifier performance) would be most affected by the adjacent hip/knee joint kinematics. We defined the gait significantly changing for a participant as 50% or more of their parameters with a statistically significant difference between PRE-NP and POST-P.

#### 2.4.2. Target Gait Parameters (MID-P and POST-NP Analysis)

The PTs also completed a session log after completing the physiotherapy session. It recorded the PT’s goals for the session, exercises administered, and desired gait effects for each of the exercises. These were used to capture and identify the specific gait parameters targeted by the PT, which are unique to each participant, to assess the classifier performance in response to MID-P and POST-NP strides. Specifically, we wanted to examine how the classifier responded depending on how these trials compared to PRE-NP and POST-P trials with a focus on the identified gait parameters, as explained in detail in the Discussion. This session log was initially developed for use with cerebral palsy, and it was adapted here for use with LLAs [42]. For each participant, the target gait parameters were selected from the PT session logs.

#### 2.4.3. Statistical Analysis

A repeated-measures ANOVA was used for each participant’s gait parameters to determine significant changes between any of the four sets of trials (*p* < 0.05). For parameters where the ANOVA indicated significant differences, post hoc Tukey-HSD was used to identify which sets of trials differed. This test is designed for multiple pairwise comparisons to avoid an increased Type I error.

## 3. Results

**Figure 5:**
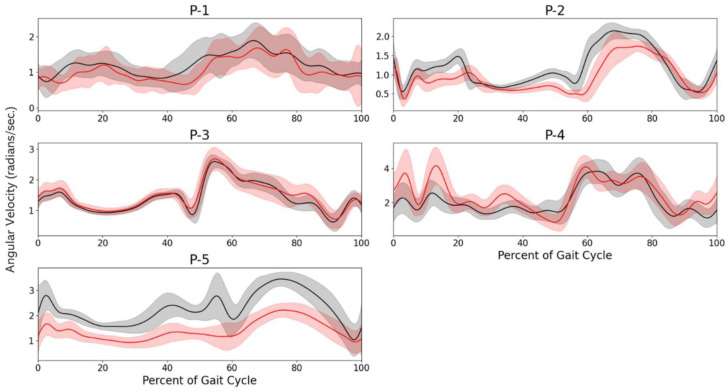
Magnitude of prosthetic-side upper-leg gyroscope signal for each of the five LLA participants (P-x = Participant x). Solid line indicates mean and the shaded area is ±1 standard de-viation. Black = PRE-NP data and Red = POST-P data.

Gyroscope signals are shown for the LLA participants in Figure 5. To assess overall variance, we averaged the standard deviations at each time point for PRE-NP and POST-P trials and calculated F-values (Table 2). The F-value is the ratio of the standard deviations. P1, P2, and P4 standard deviations were close to one (i.e., the similar variance between PRE-NP and POST-P). P3 had the highest F-value of 2.13.

### 3.1. Gait Classification Results

Table 3 shows the classification results for the LLA participants based on the five-fold cross-validation for training and test sets. Four of the participants achieved significant changes in their gait parameters (all except P3). For these participants, all trials except P4 POST-P exceeded 90% accuracy, which was the target benchmark based on the literature. The average classification accuracy for the four participants for PRE-NP was 98.65% ± 0.685 (Euclidean) and 98.98% ± 0.834 (DTW), and for POST-P was 95.45% ± 6.20 (Euclidean) and 94.18% ± 5.77 (DTW). For P3, PRE-NP classification exceeded 90% accuracy, while POST-P accuracy was 52.2% (Euclidean) and 51.7% (DTW). Based on the classification results for PRE-NP and POST-P trials across all the LLA participants, the mean difference in accuracy for the Euclidean and DTW was 0.36% ± 1.67, with the Euclidean metric being slightly more accurate than DTW at classifying PRE-NP and POST-P data. Conversely, there was a significantly higher difference between Euclidean and DTW classifier performance for MID-P and POST-NP trials across the LLA participants. The mean difference in accuracy for MID-P was 20.12% ± 16.11 and for POST-NP was 4.22% ± 7.46.

Able-bodied participant accuracy was reported as an average across the five participants in Table 4 (Control, Ankle Weight). The able-bodied participants had no significant changes in gait between any of the four sets of trials. In terms of classifier performance, PRE-NP accuracy was 51.0% (Euclidean) and 47.0% (DTW), while POST-P accuracy was 43.6% (Euclidean) and 58.3% (DTW). The mean accuracy difference for Euclidean and DTW distances was −6.0% for PRE-NP, 0.0% for MID-P, −14.7% for POST-P, and 7.0% for POST-NP.

### 3.2. Gait Parameters

To confirm that the LLA participants’ gait changed significantly during the PT session (and therefore that the classifier would be expected to recognize two gait classes), we compared PRE-NP and POST-P trials for the common gait parameters. Table 5 presents the gait parameters that had significant changes for each LLA participant. For four of the five participants, at least 50% of the parameters changed between PRE-NP and POST-P, indicating overall significant changes in their gait as a result of the gait training. Additionally, if knee and hip abduction/adduction were included for P4, 9 of 14 parameters (64%) saw significant changes between PRE-NP and POST-P. P3 had only a hip flexion/extension change and was, therefore, deemed as not having significant overall gait changes. 

Additionally, Table 6 displays gait parameter information of the targeted gait parameters for all 4 conditions.

## 4. Discussion

This work investigated a model based on time-series analysis and machine learning algorithms to classify changes in gait in LLA participating in PT-led gait training. The model was able to successfully classify between pre-training and post-training gait. Moreover, the model’s classification results for PRE-NP and POST-P corresponded to significant changes in LLA gait and non-changes in able-bodied control data. To the best of our knowledge, this work is unique in the use of time-continuous IMU signals for the classification of physiotherapy-induced changes in gait. Previous research using time-continuous signals for classification used either kinematic signals [15,23] or ground reaction forces [22]. Joint angles require at least two sensors and can be difficult to reliably measure over time using inertial sensors [43,44], while ground reaction forces require a force platform (limited to the lab) to obtain accurate force magnitudes [45]. Inertial sensors are highly flexible in their placement and configuration [6,45], and developing algorithms to leverage the data from these can support the design of compact wearable systems for gait monitoring.

### 4.1. Classification of Pre-Training (PRE-NP) and Post-Training (POST-P) Gait

Based on the literature, we aimed to exceed 90% classification [15,22]. For the four participants whose gait parameters significantly changed between PRE-NP and POST-P (P1, P2, P4, and P5), only POST-P for P4 did not meet this benchmark (with 86.6% and 87.4% accuracy for Euclidean and DTW metrics). Across those four participants, PRE-NP strides were correctly classified with an average of 98.65% ± 0.685 (Euclidean) and 98.98% ± 0.834 (DTW), while POST-P strides were correctly classified with an average of 95.45% ± 6.20 (Euclidean) and 94.18% ± 5.77 (DTW). Additionally, the model achieved a greater than 0.90 F1-score for each of the four participants, indicating a strong balance of precision and recall for identifying the gait from before and after gait training.

These promising results were achieved despite a highly diverse group of participants. Four of the five LLA participants had different amputation types and gait deviations, as well as unique gait parameter targets, functional goals, and exercises during their PT sessions. P1 was a transtibial amputee with hesitation bearing full weight on the prosthetic side, and they mostly worked on holding knee extensions during stance phases and increasing their stance time on the prosthetic side. While P5 was also working to improve prosthetic stance time, they had no knee flexion and mostly focused on core stability exercises. P4 was a bilateral amputee and worked on correcting knee valgus while symmetry was otherwise acceptable (and unchanged through the session). Our results demonstrate that it may be feasible to use minimally processed signals from a single gyroscope to assess changes in gait for a variety of different deviations and gait training targets, both spatiotemporal and kinematic. This would support the development of wearable gait analysis systems incorporating our time-series-based approach, which could assess a broad range of mobility impairments without requiring pre-tuning model parameters or pre-defining an existing set of gait parameters.

### 4.2. Classifier Performance for MID-P/POST-NP and Non-Changes in Gait

#### 4.2.1. Classification of Gait during Training (MID-P) and after PT Session (POST-NP)

For the MID-P and POST-NP gait conditions, we wanted to assess how the classifier performed based on the gait parameters in comparison to PRE-NP and POST-P conditions. For MID-P/POST-NP trials where the target gait parameters were closer to PRE-NP or POST-P, we would expect the model to classify that condition as the closer class. Conversely, if the parameters were more in between PRE-NP and POST-P, we would expect a split classification (i.e., closer to 50/50 between PRE-NP and POST-P). For these, our model achieved mixed results.

It generally performed well on the POST-NP trials. For instance, P1 regressed significantly toward the baseline in their cueing-removed trials. This can be seen in Table 6 with the green/red shaded cells. POST-NP and POST-P were significantly different for three of four parameters, while PRE-NP and POST-NP were only different for one of four parameters. With Euclidean distance, the model classified 82.6% of strides as pre-rehab strides. Additionally, Euclidean and DTW classified P2, P4, and P5 predominantly as POST-P (≥97% for all three participants). This corresponds well to results in Table 6, where POST-NP parameters differed significantly from PRE-NP and not POST-P. These results highlight the importance of developing algorithms to assess gait and retention outside the clinic. Especially for LLA with lower motor skill retention (i.e., quick regression toward baseline), such as P1; these algorithms could be incorporated into remote systems to monitor progress post-physiotherapy or guide biofeedback systems to provide LLA with the means to objectively work on gait outside the clinic.

Results were, however, mixed for MID-P strides. Target gait parameter results would suggest MID-P strides should also be classified as POST-P strides for P1, P2, and P4. This was true for P2. Conversely, for both P1 and P4, the classifier was split between PRE-NP and POST-P using Euclidean distance and was biased toward PRE-NP using DTW (72% for P1, 89% for P4). Because participant sessions were targeting many gait parameters at the same time, this made it more difficult to determine exactly where the MID-P/POST-NP conditions fell with respect to their PRE-NP (baseline) and POST-P (immediately post-training) conditions based on the target gait parameters. Furthermore, even in cases when the PT targets changed in one parameter, due to the interdependencies amongst gait parameters, other gait parameters may have exhibited significant changes (either non-target parameters and/or parameters we did not measure) [46], which could affect the gyroscope signal and consequently classifier performance. Future work should focus on more controlled experimental setups (i.e., perturbing one major gait parameter at a time) to identify the correlation between classification results and gait parameter measures. This would better allow us to determine whether the model behaves in line with clinical gait parameters.

#### 4.2.2. Classification of Non-Significant Changes in Gait Parameters

The validation on gait where significant changes did not occur also supports that the models are not over-trained/over-specific in their abilities to detect changes. Control trials with able-bodied participants showed minimal discriminatory capability from the model. For all four sets of gait trials (Table 4), the model was not much better than chance when distinguishing between data from the beginning and end of the session. Specifically, PRE-NP and POST-P trials were both within 9% of 50/50 (the result of random assignment). Additionally, one of the five LLA participants (P3) did not have significant changes in their target gait parameters or overall gait, with only 2 of the 10 common gait parameters significantly changing (Table 5). Classification results for P3 in Table 3 show that MID-P, POST-P, and POST-NP trials had mixed classification similar to the able-bodied control data. Additionally, the able-bodied participants and the LLA participant’s F1 scores were below 0.78. This is not much better than 0.67, which would be the baseline F1 score for equally distributed classes, and it helps support the assessment that our model did not achieve high precision or recall when the gait profile did not significantly change. It is unknown why PRE-NP test trials were able to be identified as PRE-NP while none of the other conditions were able to be distinguished, and further investigation is necessary to determine why this condition behaved differently. We hypothesize that this difference in results may have been due to unequal variance between the classes adversely affecting the kNN performance, specifically the POST-P trial displaying much larger variance than PRE-NP (F-value = 2.13). Because PRE-NP strides were clustered closer together, kNN could more often be able to identify PRE-NP strides based on nearest strides, even with significant overlap between the two trials. A reason for the higher variance in the POST-P trial could be because participants were practicing and learning new gait patterns during the session. Thus, they might need more time to converge on a new consistent gait profile. This difference may have been larger for P3 because they were a higher-functioning LLA whose baseline gait was well established. The impact of unequal variance is important to examine in future work classifying gait during gait training sessions where the smaller sample size (100–125 strides) could be more likely to have classes with different variances.

When developing machine learning or other signal analysis tools, interpretability and clinical relevance are both important metrics [47]. We wanted to ensure that the models were not simply picking up stochastic variation in gait since human walking does have some inherent variability; for example, over time, this can be distinguishable and classifiable as different gait patterns [48]. Our results for participants (LLA and able-bodied) whose gait parameters did not significantly change generally support that our model was responding to clinically significant changes in gait.

### 4.3. Distance Metric Comparison

The Euclidean and DTW metrics performed similarly across the participants, with an average difference of 0.30 ± 1.05% in slight favor of the Euclidean metric based on the pre-and post-rehab classification results from Table 3 for P1, 2, 4, and 5 (participants with significant changes in gait parameters). While DTW is often cited as a better metric for comparing time series, one likely factor for the lack of difference in this scenario was our segmenting and preprocessing methods. By segmenting the strides into gait cycles and normalizing their lengths, this meant that the signals being compared had standardized start and end points in relation to the signal features and a similar overall shape, thus minimizing a common drawback when using Euclidean distance to compare time series data [49]. Future work might seek to investigate different windowing methods which do not rely on gait event detection, such as sliding windows or constant time intervals, to assess dynamic time warping performance. This would allow us to apply our methods in a remote system without the need for gait event detection. This is important for developing robust algorithms that can deal with less structured data, such as that in unsupervised or uncontrolled environments, and minimize pre-processing requirements.

### 4.4. Summary of Limitations and Future Work

Future work should include alternative experiments with a more controlled intervention strategy to identify the correlation between classification results and gait parameters. This could make use of able-bodied paradigms (such as rhythmic stimulation, weight asymmetry, etc.) or gait intervention sessions that are designed to perturb one major gait parameter at a time. These protocols could allow us to more clearly assess the correlation between model performance and changes in gait profile. Future work should also assess different sensor combinations and/or the placement of the sensor(s) across a larger set of participants to identify the potential biases of locations to certain gait changes (e.g., a thigh sensor may be more sensitive to changes in the hip/upper leg than the foot, or certain locations may exhibit more or less variability, etc.) and determine the ideal sensor location(s) for a compact wearable system that is capable of classifying changes in many types of gait parameters. In addition to a larger set of participants, it would also be useful to extend testing to a broader age range to validate our model on adults and elderly LLA gait profiles and gait training activities. Lastly, the protocol should be extended to assess changes across more than one session (i.e., over longer periods of time than a single training session) and investigate continual metrics as opposed to just binary classifiers. This would also include investigation into the replacement of sensors. Because it may be a challenge to place sensors in the exact same location and orientation, developing methods that are not dependent on exact sensor placement is important for transitioning such systems from controlled experiments to real use [50,51].

## 5. Conclusions

The goal of this work was to demonstrate the feasibility of a single-sensor system that can capture changes in the overall gait profile. The study found that a single sensor can be used to classify changes in gait as a result of gait training from a PT. Combined with the results on able-bodied data, this suggests that the model could appropriately respond to significant perturbations in gait as validated by an array of conventional gait parameters. This work lays a foundation for the next steps, including the further investigation of correlations between gait parameter changes and classifier performance, the testing of different sensor locations on the body, and the exploration of continual metrics as opposed to binary classifiers. Efficient, informed rehabilitation is crucial for LLA to achieve their mobility goals and healthy gait, and this is a step toward developing objective, simple, adaptable systems which could be readily adopted in clinical practice as well as for at-home use.

## Figures and Tables

**Table 1:** LLA participant demographic information.

Participant	Age (Years)	Height (cm)	Weight (kg)	Prosthetic Type (Side)	Years Since Amputation/Years with Current Device
1	13	169	85	Transtibial (Left)	2.5/1.0
2	19	150	59	Van-Nes (Left)	10.0/0.3
3	17	179	65	Van-Nes (Left)	1.5/0.5
4	11	148	39	Transtibial (Both)	11.0/0.3
5	9	120	24	Hip Orthotic (Right)	6.0/0.1

**Table 2:** Mean standard variance (var.) for LLA participants PRE-NP and POST-P conditions. F-values (bottom row) calculated as larger variance/smaller variance.

Participant	1	2	3	4	5
Mean Var. PRE-NP	0.1799	0.0367	0.0383	0.4180	0.1978
Mean Var. POST-P	0.1861	0.0353	0.0816	0.4920	0.1136
F-value	1.0347	1.0397	2.1276	1.1770	1.7413

**Table 3:** Classification results for LLA participants. All trials (leftmost column) classified as either PRE-NP or POST-P. For PRE-NP and POST-P. Green = classifier achieved target accuracy (exceeded target benchmark of 90% for participants with significant changes or did not reach target benchmark for P3 since no significant changes). Red = did not perform as expected.

	Euclidean Distance		Dynamic Time Warping (DTW)	
**Trials**	% Classified PRE-NP	% Classified POST-P	% Classified PRE-NP	% Classified POST-P
	P1			
**PRE-NP**	0.989	0.011	1.000	0.000
MID-P	0.480	0.520	0.720	0.280
**POST-P**	0.043	0.957	0.086	0.914
POST-NP	0.826	0.174	0.652	0.348
F1 Score	0.973		0.959	
	P2			
**PRE-NP**	0.982	0.018	0.982	0.018
MID-P	0.000	1.000	0.000	1.000
**POST-P**	0.000	1.000	0.016	0.984
POST-NP	0.000	1.000	0.000	1.000
F1 Score	0.991		0.983	
	P3			
**PRE-NP**	0.911	0.089	0.918	0.082
MID-P	0.368	0.632	0.544	0.456
**POST-P**	0.478	0.522	0.483	0.517
POST-NP	0.585	0.415	0.577	0.423
F1 Score	0.763		0.765	
	P4			
**PRE-NP**	0.995	0.005	0.984	0.016
MID-P	0.500	0.500	0.889	0.111
**POST-P**	0.134	0.866	0.126	0.874
POST-NP	0.029	0.971	0.000	1.000
F1 Score	0.935		0.933	
	P5			
**PRE-NP**	0.980	0.020	0.993	0.007
MID-P	—	—	—	—
**POST-P**	0.005	0.995	0.005	0.995
POST-NP	0.029	0.971	0.029	0.971
F1 Score	0.987		0.994	

**Table 4:** Mean classification results for able-bodied participants completing control protocol with ankle weight. Green = classifier achieved target accuracy (below 90% accuracy threshold).

	Euclidean Distance		Dynamic Time Warping (DTW)	
**Trials**	% Classified PRE-NP	% Classified POST-P	% Classified PRE-NP	% Classified POST-P
**PRE-NP**	0.774	0.226	0.699	0.301
MID-P	0.286	0.714	0.286	0.714
**POST-P**	0.221	0.779	0.285	0.715
POST-NP	0.380	0.620	0.448	0.552
F1 Score	0.776		0.705	

**Table 5:** Common gait parameters. P-value results of Tukey-HSD significance test on common gait parameters PRE-NP vs. POST-P for LLA participants. Dash = non-significant difference. N/A for P5 = not applicable due to fixed-knee prosthetic (no flexion allowed). * For P4, number of parameters with changes increases to 9/14 (64%) if target parameters knee and hip abduction/adduction are included.

Gait Parameter	P1	P2	P3	P4	P5
Stance-Time Symmetry Ratio	<0.001	–	–	0.005	<0.001
Stance Time Pro.	<0.001	<0.001	–	–	–
Stance Time Non-Pro.	–	<0.001	–	0.005	<0.001
Double Stance Support	<0.001	<0.001	–	–	<0.001
Step Length Pro.	<0.001	<0.001	–	–	<0.001
Step Length Non Pro.	–	0.02	–	–	<0.001
Knee Flex/Ext Pro.	–	<0.001	–	<0.001	N/A
Knee Flex/Ext Non-Pro.	–	–	–	–	<0.001
Hip Flex/Ext Pro.	<0.001	<0.001	<0.001	<0.001	<0.001
Hip Flex/Ext Non Pro.		<0.001	<0.001	0.01	<0.001
Number of Parameters with Significant Changes	5/10	8/10	2/10	5/10 *	8/9

**Table 6:** Main targeted gait parameters for amputee participants (mean ± standard deviation) based on the session log data. Letters denote pairs where there is significant difference between parameters for those two trials, *p* < 0.05. Cells with red coloring indicate parameters significantly different from POST-P for that participant, cells with green coloring = significantly different from PRE-NP. — in P5 data were not available for MID-P as it could not be collected due to a shorter than normal session and not reported for knee flexion/extension because their prosthetic prevented knee flexion (i.e., locked). Abbreviations in the table: Abd/Add = Abduction/Adduction, Flex/Ext = Flexion/Extension, and Pro = Prosthetic. All kinematic parameters reported as range of motion (ROM).

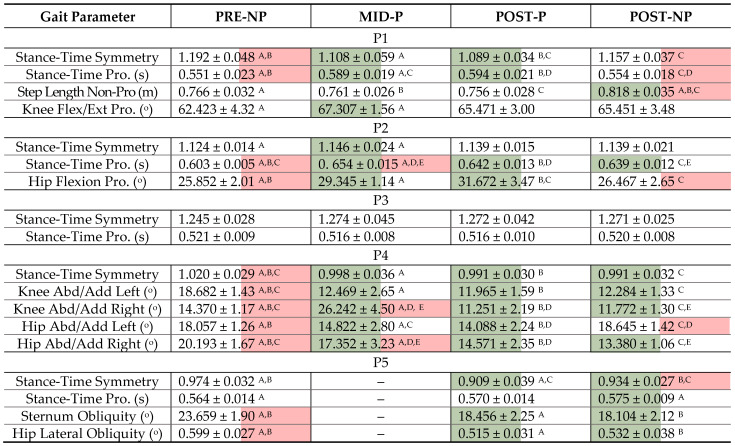

## Data Availability

The data presented in this study are available on request from the corresponding author. The data are not publicly available due to ethics restrictions on data dissemination and storage.

## Abbreviations

The following abbreviations are used in this manuscript:

LLA	Lower-Limb Amputee
ML	Machine Learning
PT	Physiotherapy/Physiotherapist
DTW	Dynamic Time Warping
kNN	k-Nearest Neighbors
PRE-NP	PRE-gait training session, No Physiotherapist cueing
MID-P	MIDpoint of gait training session, Physiotherapist cueing
POST-P	POST-gait training session, Physiotherapist cueing
POST-NP	POST-gait training session, No Physiotherapist cueing
